# β-Synuclein as a candidate blood biomarker for synaptic degeneration in Alzheimer’s disease

**DOI:** 10.1186/s13195-022-01125-1

**Published:** 2022-11-30

**Authors:** Pablo Mohaupt, Marie-Laure Pons, Jérôme Vialaret, Constance Delaby, Christophe Hirtz, Sylvain Lehmann

**Affiliations:** 1 LBPC-PPC, Université de Montpellier, IRMB CHU de Montpellier, INM INSERM, Montpellier, France; 2 Sant Pau Memory Unit, Hospital de la Santa Creu i Sant Pau - Biomedical Research Institute Sant Pau - Universitat Autònoma de Barcelona, Barcelona, Spain

**Keywords:** β-Synuclein, Alzheimer’s disease, Blood biomarker, Synaptic dysfunction

## Abstract

Synaptic degeneration is an early event closely associated with the course of Alzheimer’s disease (AD). The identification of synaptic blood biomarkers is, therefore, of great interest and clinical relevance. The levels of most synaptic proteins are increased in the cerebrospinal fluid (CSF) of patients with AD, but their detection in blood is hitherto either unavailable or not very informative. This paradigm is related to their low concentration, their peripheral origin, or the presence of highly abundant blood proteins that hinder detection. In recent years, significant progress has been made in detecting the presynaptic protein β-synuclein. This mini-review summarizes the results that highlight the role of β-synuclein as a candidate blood marker for synaptic degeneration in AD.

## Background

**Fig. 1:**
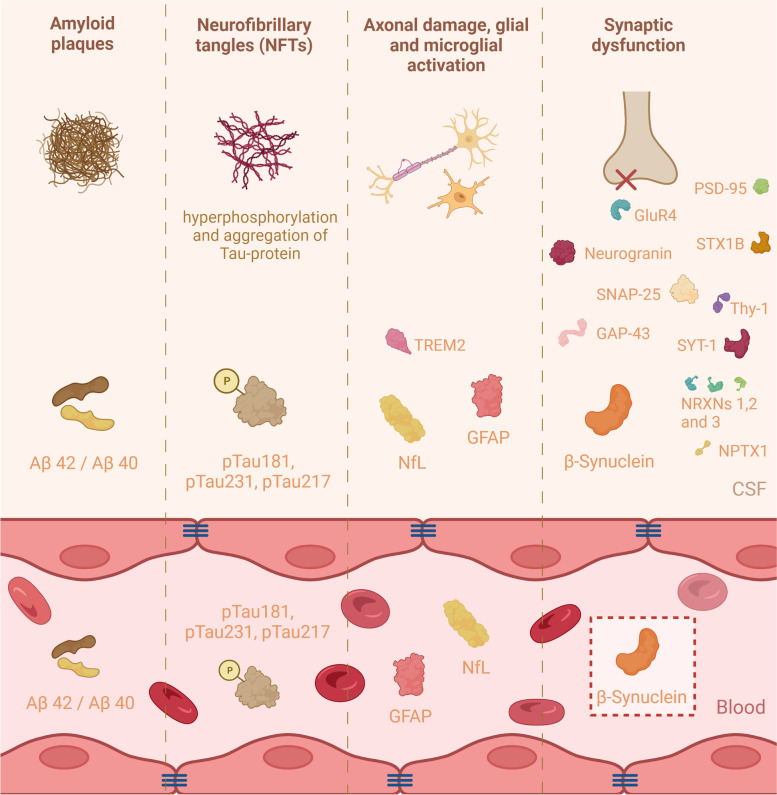
Pathophysiological hallmarks of Alzheimer’s disease and its associated biomarkers in either blood or cerebrospinal fluid. Alzheimer's disease is characterized by amyloid plaques, neurofibrillary tangles, axonal damage, microglial activation, and synaptic dysfunction. These pathophysiological hallmarks are responsible for significant modification of CSF Amyloid-β42/40 ratio, levels of tau with abnormal phosphorylation at positions 181, 231, and 217, neurofilament light chain (NfL), and glial fibrillary acidic protein (GFAP) levels. These variations are also detected in the blood of AD patients. The levels of numerous proteins involved in synaptic function vary in CSF in (preclinical) AD. Proteins originating from the central nervous system are often present in low concentrations that are difficult to detect. A fraction of synaptic proteins is also expressed in other tissues and are therefore not suitable as specific blood biomarkers for synaptic dysfunction. To date, β-synuclein, whose expression is brain-specific, is the only synaptic protein that is determined to be significantly increased in the blood of AD patients

The etiology of Alzheimer’s disease (AD) remains controversial, despite recent developments in genetics and cell biology. AD is characterized by neuronal loss, extracellular amyloid-β plaques, and neurofibrillary tangles consisting of strings of hyperphosphorylated tau located within neurons [1]. Until recently, only therapies were available to treat the symptoms of AD, and clinical trials building on the amyloid cascade theorem struggled to halt or delay disease progression [2, 3]. Anti-tau antibodies have so far been unsuccessful, whereas the anti-amyloid-beta antibody aducanumab was approved by the Food and Drug Administration (FDA). This treatment is supposed to target the underlying disease process of AD, while its effectiveness is moderate but controversial [4]. Thus, there is still a need for more effective or targeted approaches and to unravel the pathophysiological subtypes of AD [5]. As treatments become available, there is also a clinical need for biomarkers that detect AD in its earliest stages and predict and monitor disease progression [6]. Synaptic degeneration is the neuropathological hallmark most highly correlated with cognitive impairment in AD; hence, the interest in synaptic biomarkers that may complement the amyloid and tau panels already in use (Fig. 1) [7]. Synaptic degeneration in AD can already be determined by quantifying post- and presynaptic proteins in CSF and by measuring the levels of SV2A with positron emission tomography (PET) [8, 9]. The measurement of brain-specific synaptic proteins in blood is currently not available but can be of great relevance for clinical trials, disease monitoring, and early diagnosis. Here, we summarize recent findings and outline the rationale that the presynaptic protein β-synuclein is an early and prognostic blood marker for AD.

## β-Synuclein in Alzheimer´s disease

β-Synuclein is a presynaptic protein encoded by the synuclein Beta locus (SNCB) and is predominantly expressed in the brain. β-Synuclein forms, together with α-synuclein and γ-synuclein, a group of proteins dubbed “Synucleins” and is shown to inhibit α-synuclein aggregation [10]. α-Synuclein is extensively researched to assess its biomarker potential in synucleinopathies such as Parkinson’s disease (PD), dementia with Lewy bodies (DLB), and multiple system atrophy (MSA), which are characterized by abnormal accumulation of α-synuclein aggregates [11]. As such, the presence of elevated β-synuclein in the CSF of AD patients was first detected serendipitously in an endeavor to identify abnormalities specific to a particular synucleinopathy [12]. A significant increase with a high correlation with CSF tau was observed in AD and Creutzfeldt-Jakob disease (CJD). Of note, β-synuclein was slightly higher in dementia with DLB and PD-related dementia compared with controls and PD without dementia, suggesting that β-synuclein may be more associated with cognitive impairment than with motor impairment. The increase of β-synuclein in AD CSF was also observed in a study targeting synaptic proteins with mass spectrometry and was confirmed in an immunodetection study [13, 14]. The latter suggests that β-synuclein is altered more specifically in amyloidopathies, as no changes were observed in non-amyloid pathologies such as frontotemporal dementia (FTD). Furthermore, the increase was already present in prodromal patients with mild cognitive impairment (MCI-AD), highlighting its potential as an early diagnostic and prognostic biomarker candidate. The AD-specific increase was also observed in a study researching synaptic dysfunction in different neurodegenerative diseases [15]. A major step in assessing the value of β-synuclein as a biomarker for AD was the development of an immunoprecipitation mass spectrometry (IP-MS) method that can be applied in both CSF and blood [16]. This novel assay confirmed the previous results obtained in CSF and, in particular, showed that the levels of β-synuclein were also significantly higher in the blood of AD patients. This study positioned β-synuclein as the first synaptic blood biomarker for AD. The increase in MCI-AD was more moderate and did not reach significance, but this is likely due to the small cohort size. The increase was also confirmed in a study in adults with Down syndrome (DS), who are at high risk of developing AD due to overexpression of the APP gene located on chromosome 21 [17]. Of particular note, β-synuclein was already altered in preclinical AD and was elevated earlier than pTau181, confirming its early diagnostic and prognostic interest. More recently, blood β-synuclein levels were found to correlate with cognitive impairment and brain atrophy in AD, which indicates its association with disease severity [18].

The IP-MS analytical approach is innovative but requires large blood sample volumes and has low turnover, hindering the analysis of large cohorts and routine use. Recently, an ultrasensitive immunodetection assay with Simoa was developed to target β-synuclein in blood [19]. So far, this test has been applied to CJD and it has yet to be determined if this assay can match the clinical performance of IP-MS in AD-related cohorts.

There is no comparison of blood β-synuclein with other synaptic markers such as SNAP-25, SYT-1, neuronal pentraxins (NPTXs), or neurogranin (Ng). Indeed, the presynaptic SYT-1 and SNAP-25, the postsynaptic Ng, and NPTXs which are present in both pre- and post-synapses all show altered levels in CSF [20–23]. Nonetheless, most synaptic proteins are not yet assayable in the blood, even with ultrasensitive methods as tested recently for SNAP-25 [24]. Ng can be detected in blood, but its expression is not restricted to the brain; therefore, the changes observed in CSF are not specifically reflected in the blood [9].

The combined results outlined in this mini-review show that β-synuclein could potentially complement the panel of blood biomarkers for AD as a marker for synaptic degeneration, whereas phosphorylated tau biomarkers reflect amyloidopathy and tau-pathology.

## Conclusion

In conclusion, β-synuclein is to date the only detectable blood biomarker for synaptic degeneration in AD. The switch from mass spectrometry to an immunodetection method increases the accessibility of β-synuclein quantification in larger research cohorts. Its value as a marker for AD will have to be tested in cohorts and compared with blood markers such as pTau217 and pTau231, whose performances remain unmatched to date [25].

## Data Availability

Not applicable.

## Abbreviations

Aβ40: Amyloid-β 40
Aβ42: Amyloid-β 42
AD: Alzheimer’s disease
CJD: Creutzfeldt-Jakob disease
CSF: Cerebrospinal fluid
DS: Down syndrome
FDA: Food and Drug Administration
GAP-43: Axonal membrane protein GAP-43
GFAP: Glial fibrillary acidic protein
GluR4: Glutamate receptor 4
IP-MS: Immunoprecipitation followed by mass spectrometry
MCI-AD: Mild cognitive impairment due to AD
NfL: Neurofilament light chain
Ng: Neurogranin
NPTX1: Neuronal pentraxin-1
NPTXs: Neuronal pentraxins
NRXN: Neurexin
PD: Parkinson’s disease
PET: Positron emission tomography
PSD-95: Postsynaptic density protein 95
pTau181: Tau with phosphorylation at positions 181
pTau217: Tau with phosphorylation at positions 217
pTau231: Tau with phosphorylation at positions 231
SNAP-25: Synaptosomal-associated protein 25
SNCB: Synuclein Beta locus
SV2A: Synaptic vesicle glycoprotein 2A
STX1B: Syntaxin-1B
SYT-1: Synaptotagmin-1
Thy-1: Thy-1 membrane glycoprotein
TREM2: Triggering receptor expressed on myeloid cells 2
